# Determinants of Self-Reported Unmet Healthcare Needs by Disability Status: A Secondary Cross-Sectional Analysis of Linked National Survey and Administrative Data from Korea

**DOI:** 10.3390/healthcare14121748

**Published:** 2026-06-17

**Authors:** Boram Lee

**Affiliations:** National Rehabilitation Center, Seoul 01022, Republic of Korea; brl9191@korea.kr; Tel.: +82-10-8901-1460

**Keywords:** health services needs and demand, healthcare disparities, persons with disabilities, health services accessibility, socioeconomic factors, sex factors, Korea

## Abstract

Background/Objectives: People with disabilities face a disproportionately higher disease burden alongside reduced healthcare accessibility, resulting in elevated unmet healthcare needs (UHN). Understanding the factors that drive UHN—and whether these factors differ by disability status—is critical for developing targeted public health interventions. Methods: A secondary cross-sectional analysis was conducted using linked national survey and administrative data—specifically the Korea National Health and Nutrition Examination Survey (KNHANES) cycles VI to VIII (2013–2021) linked with National Health Insurance Service (NHIS) administrative records—to examine determinants of healthcare access and utilization barriers by disability status. Independent variables were selected based on Andersen’s Behavioral Model of Health Services Use for vulnerable populations, encompassing predisposing factors, enabling factors, and need factors (including functional status indicators). Interaction terms between disability status and sex, and between disability status and household income level, were introduced to identify effect modification by disability status. Results: People with registered disabilities had significantly higher UHN compared to those without disabilities. The contributing factors to UHN differed between the two groups, with sex and household income showing statistically significant interaction effects with disability status, indicating that their associations with UHN vary depending on whether an individual has a registered disability. Conclusions: UHN in people with disabilities is shaped by a distinct set of determinants compared to the general population. The overall pattern of contributing factors differed between people with and without disabilities across multiple dimensions. These findings highlight the need for tailored healthcare policies that account for the unique vulnerabilities of people with disabilities, rather than applying uniform strategies across all population groups.

## 1. Introduction

Access to healthcare services when needed is a fundamental component of any well-functioning health system and a cornerstone of universal health coverage (UHC) [1]. Measuring whether individuals receive the healthcare they need is essential for evaluating system performance and identifying gaps in service delivery [2]. Self-reported unmet healthcare needs (UHN)—defined as situations where individuals perceive a need for care but do not receive it—are widely used as a key indicator of healthcare access and utilization barriers [3,4,5,6]. Understanding the determinants of UHN across different population groups is therefore critical for informing targeted healthcare policies for high- risk groups with poor access. People with disabilities represent one such high-risk group, facing a disproportionate burden of disease alongside well-documented barriers to healthcare access [7,8,9,10]. Health disparities between people with and without disabilities have been described in various studies, where differences are found in health-related risk factors and health outcomes [4]. Difficulties in healthcare access further widen this health gap [9,10], and higher UHN of people with disabilities compared to the rest of the population have been reported previously [8,11,12].

Despite the clear burden of UHN in the disability population, significant gaps remain in the health services research literature. Studies systematically examining which predisposing, enabling, and need factors drive UHN in the disability population—and whether these determinants operate differently compared to the general population—are limited [13]. Whether disability itself constitutes an independent determinant of UHN beyond these factors is also insufficiently addressed. A recent Canadian study found that disability remained a significant factor for UHN after adjusting for sociodemographic variables [14], but the complexity of health, functional, and social factors accompanying disability status warrants further verification across different settings and with a more comprehensive set of covariables [15]. From a service delivery perspective, interventions addressing inequitable access to healthcare appear not to have reached the disability population effectively, as their elevated UHN has remained consistent over the years in Korea and elsewhere [16,17]. This persistent gap suggests that a distinct, evidence-based approach to healthcare service planning for the disability population is needed—one grounded in a systematic analysis of their specific determinants of UHN.

Andersen’s Behavioral Model of Health Services Use, and its extension to vulnerable populations by Gelberg, Andersen, and Leake (2000), provides a well-established conceptual framework for understanding how predisposing, enabling, and need factors shape healthcare utilization and access—particularly among marginalized groups [18,19]. People with disabilities fit well within this vulnerable population framework, as they are disproportionately affected across all model components. Predisposing factors include individual sociodemographic characteristics, such as age, sex, marital status, household size, and educational level. Disability status may also be included as a predisposing vulnerability factor. Enabling factors, including household income, employment status, and medical insurance type, reflect the resources available to access healthcare services. Need factors encompass both perceived and evaluated health needs, including subjective health status and chronic conditions. Functional status indicators, such as functional limitations and mobility problems were also included, given that physical and environmental accessibility is particularly important in healthcare utilization for people with disabilities [7,8,20,21]. While need factors act directly on healthcare utilization, predisposing and enabling factors may have a more complex and differential impact on utilization in vulnerable populations with complex backgrounds, such as the disability population.

In this study, we first aim to identify possible contributing factors to self-reported unmet healthcare needs in people with disabilities and explore whether disability itself could be a significant factor for UHN. Interaction terms between disability status and the most prominent predisposing and enabling factors—sex and household income levels—will be introduced to investigate whether the associations between contributing factors and self-reported UHN are moderated by disability status. Further stratification analysis based on disability status will be conducted to identify factors that operate differently with self-reported UHN in people with disabilities compared to those without disability.

## 2. Materials and Methods

### 2.1. Data Source and Study Population

**Figure 1:**
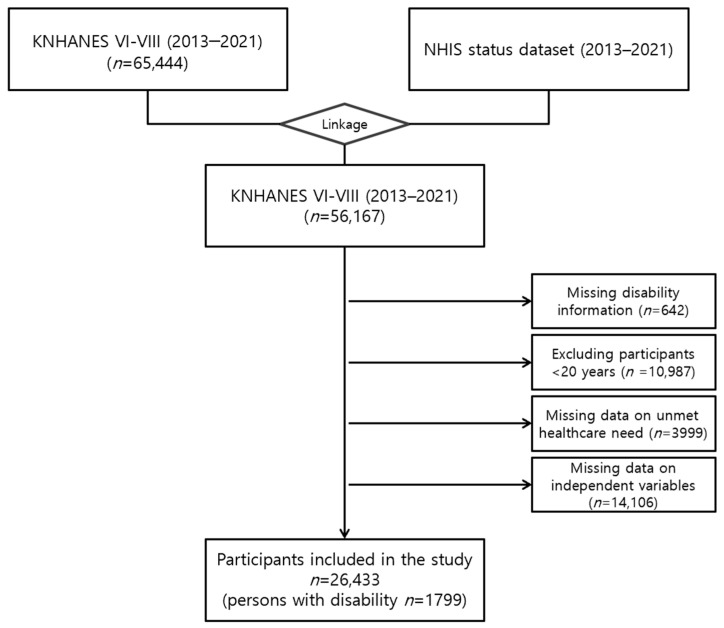
Flow diagram describing study participation.

This study performed a secondary data analysis using a special dataset prepared from the Korea National Health and Nutrition Examination Survey (KNHANES) cycles VI to VIII, conducted from 2013 to 2021. KNHANES is an annually performed cross-sectional study from a nationally representative sample that serves the purpose of assessing the health and nutritional status of the national population [22]. It includes a wide range of data from sociodemographic status and health risk factors to health utilization, general health, health-related quality of life, chronic disease profiles, and dietary status of the respondents. Since KNHANES originally did not include disability status information of the respondents, the health information of people with disabilities was not captured in the survey. Disability status information was incorporated into the dataset through a data linkage process via the Korea Healthcare Bigdata Platform using the 2013–2021 National Health Insurance Service (NHIS) insured status dataset. The Korea Healthcare Bigdata Platform is operated by the Korea Health Information Service and the Ministry of Health and Welfare, through which datasets are provided following a pre-approval process and authorized access. While the original KNHANES datasets are made publicly accessible for research purposes, the datasets used in this study were accessible only through pre-authorization in accordance with their data access policy. The KNHANES data included 65,441 individuals in total, and the corresponding NHIS data were matched using linkage keys provided by a trusted third party for each identified individual. The linked data resulted in an 85.8% linkage success rate, producing a dataset of 56,167 observations (Figure 1). As a result, the study population included the survey population of KNHANES VI to VIII with disability status added to the original dataset. Among them, those without confirmed disability information, people under 20 years of age, and those missing outcome and independent variables were excluded, resulting in 26,433 individuals in total.

### 2.2. Variables and Measures

#### 2.2.1. Dependent Variables

Self-reported UHN was measured by the question “During the previous 12 months, was there a time when you felt you needed healthcare services but did not receive them?” in the KNHANES health questionnaire. The respondents answered either “yes” or “no” according to their experiences, and those who answered “yes” were classified as having UHN.

#### 2.2.2. Independent Variables

Independent variables were selected based on previous studies and Andersen’s Behavioral Model of Health Services Use for vulnerable populations [18,23,24,25], encompassing predisposing factors, enabling factors, and need factors, with disability status as the primary exposure variable and a predisposing vulnerability factor. Predisposing factors included age (20–34, 35–49, 50–64, 65–79, ≥80 years), sex, household size (1, >1), marital status (married, other), and educational status (primary, lower-secondary, higher-secondary, tertiary). Sex refers to biological sex as recorded in the KNHANES survey data, categorized as male or female in this study. Enabling factors were household income (1st quartile, 2nd quartile, 3rd quartile, 4th quartile), paid work (yes, no), and medical insurance status (National Health Insurance, Medicaid). Need variables included subjective health status (very bad, bad, moderate, good, very good), obesity (no, yes), hypercholesterolemia (no, yes), diabetes (no, prediabetes, diabetes), and hypertension (no, prehypertension, hypertension). Obesity was defined as BMI ≥ 25 kg/m^2^, consistent with WHO recommendations for Asian populations. Hypertension classification followed the KNHANES survey categorization: no hypertension (systolic BP < 120 mmHg and diastolic BP < 80 mmHg), prehypertension (systolic BP 120–139 or diastolic BP 80–89 mmHg), and hypertension (systolic BP ≥ 140 mmHg or diastolic BP ≥ 90 mmHg). While the term ‘prehypertension’ is no longer used in current ACC/AHA guidelines, it has been retained here to accurately reflect the original KNHANES data classification. Considering the functional relevance in the care of people with disabilities, three need variables representing functional status were added to the model: the presence of functional limitations (yes, no), levels of problems in mobility (no problem, some problem, extreme problem), and levels of problems in usual activities (no problem, some problem, extreme problem). Disability status was defined by the national disability registration criteria, as the information was derived from the Korea National Disability Registration System. Disability is assessed based on medical criteria and is largely divided into physically and mentally disabled, with further classifications leading to 15 types—physical, brain-lesion related, intellectual, auditory, visual, facial, communicative, kidney, respiratory, liver, cardiac, psychosocial, autistic, epilepsy, and stoma disability. The details of the assessment and registration criteria are described elsewhere [26].

### 2.3. Statistical Analyses

Statistical analyses were performed using SAS software version 9.4 (SAS Institute Inc., Cary, NC, USA). Descriptive statistics were used to describe the sociodemographic, health status, and functional variables in terms of frequency and percentages. A chi-square test of independence was performed to identify differences in unmet healthcare needs according to the different levels of the given variables. Multiple logistic regression analyses were performed to estimate the odds ratios of various factors on self-reported unmet healthcare needs. Three models were constructed: the first model included predisposing and enabling (sociodemographic) factors; the second model additionally included the complete set of variables. In addition, the analyses were stratified by disability status to evaluate whether the associations between independent variables and self-reported unmet healthcare needs are modified by disability status. Effect modification was also assessed by including interaction terms in the multiple logistic regression analyses. Variables were selected based on the previous empirical literature on UHN and forced entry approach in all regression models. Missing responses were handled by the KNHANES guideline, and different responses based on the items, such as “unknown” and “not relevant”, were classified as a missing response. For the analysis, missing data were handled by using complete case analysis, consistent with standard practice of large nationally representative surveys. To assess potential multicollinearity among independent variables, especially among the functional variables, Variance Inflation Factor (VIF) diagnostics were computed for all variables in the final model. VIF ranged from 1.11 to 2.64, well below the conventional threshold of 10, indicating no evidence of problematic multicollinearity. Statistical significance was set at *p* < 0.05. Given the large, nationally representative sample size of 26,433 individuals, the study was considered adequately powered to detect statistically significant associations between the variables of interest. However, the disability group (*n* = 1799) may be underpowered for certain stratified analyses, and findings from theses analyses should be interpreted with appropriate caution.

### 2.4. Ethical Considerations

This study was conducted in accordance with the Declaration of Helsinki and was approved by the Institutional Review Board of the National Rehabilitation Center, Korea (IRB No. NRC-2023-05-036). Exemption from full review was granted due to the use of previously collected and anonymized data.

## 3. Results

### 3.1. General Characteristics and Unmet Healthcare Needs of the Study Population

Among the 26,433 individuals, 24,634 (93.2%) did not have disabilities and 1799 (6.8%) had disabilities (Figure 1). In total, 2477 (10.3%) reported having unmet healthcare needs.

The sociodemographic and clinical characteristics of the study population by disability status are presented in Table 1. Individuals with and without disabilities showed different distributions of unmet healthcare needs and sociodemographic, health, and functional characteristics. Respondents with disabilities had a higher rate of unmet healthcare need (13.2% vs. 10.1%). They had a higher proportion of men (61.5% vs. 46.7%), individuals over 50 years of age (84.5% vs. 52.0%), those who were married (91.4% vs. 83.7%), and single-person households (18.6% vs. 10.4%). In terms of socioeconomic status, people with disabilities had a significantly higher rate of Medicaid coverage (13.1% vs. 2.4%), and a higher proportion in the lowest quartile of household income (41.9% vs. 16.5%) and lowest level of education (45.6% vs. 19.1%). People with disabilities also had lower participation in paid work (44.9% vs. 63.3%). People with disabilities showed poorer health, including a higher proportion reporting bad or very bad subjective health (38.4% vs. 17.2%), higher prevalence of obesity (41.8% vs. 34.4%), and higher morbidity of hypertension (49.8% vs. 31.1%), diabetes (25.8% vs. 11.1%), and hypercholesterolemia (27.3% vs. 20.3%). With regard to functional factors, respondents with disabilities had significantly more functional limitations, and more frequent problems in mobility (38.4% vs. 12.1%) and usual activities (37.6% vs. 6.6%).

The self-expressed unmet healthcare needs were different for each category of most of the predisposing, enabling, and need factors and the distribution were also different by the disability status for some variables (Table 2). For instance, female sex showed higher rate of unmet healthcare need, but the percentage was much higher in those with disability. And obesity showed higher unmet healthcare need in the disability group but not in those without disability.

### 3.2. Association Between Sociodemographic, Health, and Functional Factors and Unmet Healthcare Needs

The first multiple logistic regression model included predisposing and enabling factors—disability status, sex, age, marital status, household size, insurance type, household income, education, and paid work—all of which showed significant associations with UHN (Table 3). Model 2 included all variables; statistically significant associations with UHN were found for most variables, except for disability status, insurance type, obesity, diabetes, and hypercholesterolemia. The final model showed that female sex, younger age, married status, living alone, lower household income, lower educational attainment, unemployment, poor subjective health, absence of hypertension, functional limitations, and mobility and usual activity problems were significantly associated with UHN.

Interaction terms between disability status and sex, and between disability status and household income, were statistically significant. Among people with disabilities, the association between sex and UHN was stronger (OR = 1.61), while the association between household income and UHN was weaker (OR = 0.58) (Table 3). This is consistent with Table 4, where stratum-specific odds ratios for each categorical level differ for these two variables.

### 3.3. Association Between Sociodemographic, Health, and Functional Factors and Unmet Healthcare Needs by Disability Status

The associations between predisposing, enabling, and need factors and UHN differed by disability status. In those with disability, the association between sex and UHN was stronger, with female sex showing a higher odds ratio among people with disabilities. The stronger association between younger age and UHN observed in respondents without disability was not evident in those with disability. Married status and living alone were both significantly associated with higher odds of UHN in those without disability, but not in the disability group. Insurance type was not significantly associated with UHN in either group, although Medicaid showed borderline significance in respondents with disability. Household income and education both had significant associations with UHN in the non-disability group, with lower income and lower education showing higher odds of UHN. These relationships were not significant in the disability group, and lower income tended to be associated with lower odds of UHN, contrary to the pattern observed in those without disability. Poor subjective health status was associated with UHN in both groups, but the point estimates were larger in the disability group, though with considerable imprecision due to the modest sample size of the disability group. Having hypertension was significantly negatively associated with UHN in the non-disability group, and this was true for diabetes in the disability group. Functional limitations and mobility problems were positively associated with UHN in both groups, but with higher odds in the disability group. Usual activity problems were associated with higher odds in the non-disability group but showed non-significance in the disability group. Although these were statistically significant findings, it should be noted that several estimates in the disability subgroup are based on small cell sizes and show wide confidence intervals, which warrants cautious interpretation of the findings.

## 4. Discussion

Applying Andersen’s Behavioral Model of Health Services Use for vulnerable populations [18,19] as a guiding framework, this study provides new evidence on how predisposing, enabling, and need factors collectively drive self-reported UHN in the disability population—and how these contributions differ from those without disability. People with disabilities were found to bear higher proportions of health-related risk factors and were more likely to have UHN, consistent with the broader health services literature [12,21,23,27]. Importantly, the relative contributions of each model domain varied meaningfully by disability status, which bear implications for how healthcare services should be planned and delivered for this population.

Surprisingly, disability itself was not a significant factor for UHN after adjusting for the sociodemographic, health, and functional variables, suggesting that vulnerability to UHN in people with disabilities lies in the complexity of their contributing factors rather than disability status per se. Within the Andersen framework, this suggests that people with disabilities experience a distinct configuration of predisposing, enabling, and need factors that collectively drive their elevated UHN, rather than disability acting as an independent determinant. A recent study from Canada found disability as a significant factor for UHN but used a model that only included sociodemographic factors [15]. This is consistent with our study, where a significant association between disability and UHN was found when only sociodemographic factors were considered. It should also be considered that functional limitations may mediate the effect of disability status on UHN. Within the Andersen model framework, functional limitations are classified as need factors that directly drive healthcare utilization. Since disability status is closely associated with functional limitations, it is possible that the effect of disability on UHN is mediated through these functional factors, such that disability loses independent significance once they are accounted in the model. Future studies are warranted to clarify such mediation.

Obesity and hypercholesterolemia were not associated with UHN, and having hypertension in the non-disability group and diabetes in the disability group were associated with lower UHN. Although chronic diseases are generally known to be associated with higher UHN [9], our study produced contrary findings. Within the need domain of the Andersen model, this may be understood in terms of access to a regular source of healthcare. Having a regular source of healthcare has been shown to reduce the likelihood of unmet healthcare needs in previous studies [15,27]. Korea has a high treatment rate for hypertension and diabetes (74% and 70%, respectively) [28,29,30], and those using health services in this process may benefit by addressing their health needs through regular visits. Since most hypertension and diabetes care takes place in the primary care setting [30], regular health checkup services for high-risk patients may be an effective way to address their needs.

Another distinctive finding was that the effect of various factors on UHN differed depending on disability status, consistent with the vulnerable populations’ extension of the Andersen model, which posits that the relative weight of predisposing, enabling, and need factors may differ among vulnerable subgroups [18,19]. This was shown across many variables included in the study—sex, age, marital status, household size, household income, education, morbidity of hypertension and diabetes, and problems in usual activities. In addition, statistically significant effect modification by disability status was found for sex and household income.

Female sex, a key predisposing factor in the Andersen model, has been previously reported as a predictor of higher UHN due to various reasons including social vulnerabilities and additional roles of housework or childcare [23,24,31]. Other studies regarding disability populations reported women with disabilities having more difficulties in healthcare utilization than their male counterparts [14,15,32]. The United Nations also reported that women with disability are three times more likely to have unmet needs for healthcare compared to men without disability, due to intersecting discrimination and barriers [11]. This was also true in our study, where women with disability had nearly three times higher UHN than men without disability (20.2% vs. 7.6%). Not only was the UHN gap between the subgroups evident, but the multiplicative effect of female sex and disability on higher UHN was also demonstrated. This justifies more targeted actions for this vulnerable subgroup. This is noteworthy since, to our knowledge, no previous study has analyzed the differential effect of sex or gender on UHN according to disability status.

Household income, a central enabling factor in the Andersen model, is a well-known determinant of healthcare access and UHN [8,16,24,27,33]. Results in the non-disability population were consistent with this, showing increasing odds of UHN with lower household income levels. However, this was not evident in the disability group, where although not statistically significant, lower odds of UHN were found in the two lowest quartiles of household income (Table 4). The moderated effect of income level by disability status shown in Table 3 supports the notion that enabling factors operate differently in the disability population. A recent national disability survey from Korea reported that transport was the most important factor for UHN in people with disabilities, and not having time and needing a companion followed as the most frequent reasons [16]. This is different from the general population, where not having time, financial burden, and distance were the most common reasons [1,34]. This pattern is further supported by a recent study of a Korean community spinal cord injury population, where long-distance travel and public transportation were more problematic than financial barriers for activity and participation [20]. We may hypothesize through these findings that in the Korean disability population, physical accessibility and transportation barriers rather than financial enabling factors may be more critical to healthcare access. However, as no direct measures of transportation and physical access were included in the study, this interpretation remains speculative and requires further studies including the physical access measures. Furthermore, it has to be considered that the medical and social care subsidies for registered persons with disabilities in Korea, some limited to those with lower income levels, may have partially offset financial barriers to healthcare access. This may have affected and attenuated the UHN gradient across income levels in the disability group. These findings suggest that the household income factor operates through different mechanism in the disability population, and that policy efforts focused solely on addressing financial barriers may be insufficient without concurrent improvements in physical accessibility and transport support. Also, health services that confines its recipients to the lowest household income level, should be widened to all income levels in the disability population, as level of income does not predict the unmet need for service use.

Although statistically significant effect modification was not demonstrated for these variables, the differential effects observed across disability strata are worth noting for further insights. In terms of predisposing factors, younger age was found to be associated with higher odds of UHN in the general and non-disability population, consistent with several previous studies [15,23,24,25]. Although older age is associated with increased health-related risk factors and healthcare needs [35], younger individuals may have less time and fewer resources available for healthcare utilization, hold higher expectations or different perceptions of healthcare, or lack information and knowledge on healthcare resources [23,25,36]. Interestingly, in the disability group this tendency was less evident, with all age groups showing similarly elevated odds, particularly between 50 and 80 years. This may reflect lower resources and information relative to the high burden of poor health across all age groups in the disability population. Other predisposing factors—marital status, household size, and educational level—did not show significant associations with UHN in those with disability, whereas people without disability showed significant associations. Although social support is considered important in determining UHN [37], these predisposing factors appear less relevant in the disability population compared to enabling and need factors. While primary educational level was significantly associated with UHN in people without disability, no significant association was found in the disability group. This may suggest lower relevance of this predisposing factor in the population, although it has to interpreted with caution.

Regarding need factors, poor subjective health was associated with UHN in both disability and non-disability populations, consistent with previous findings across different study populations [23,25]. Poor health implies greater healthcare needs, and this increased burden may lead to a higher likelihood of UHN. Furthermore, poor health itself may make it more difficult to seek and access services. Whatever the reasons, timely access and use of healthcare among those with higher needs is critical in achieving health equity and better health outcomes [5]. In terms of functional need factors, difficulty in mobility was strongly associated with UHN in both groups, while usual activity problems were not associated with UHN in the disability group. This suggests that in this population, physical access and transport barriers may be more critical determinants of UHN than restrictions in daily activities. Taken together with the household income findings, these results suggest that physical accessibility may be a more critical determinant of UHN than financial accessibility in the disability population, distinguishing it from the general population [8].

This study has several limitations. First, the study did not examine disability types separately and therefore could not identify which type is most vulnerable to UHN. Addressing this could help improve the tailoring of healthcare use strategies, given the great heterogeneity among disability types [8,9,12,38]. People with sensory disabilities such as auditory and visual disabilities may face communication difficulties and may benefit from improved information accessibility, whereas those with physical disabilities mostly experience mobility barriers which require accessible facilities and devices as well as transportation. People with developmental and mental disabilities are known to face cognitive barriers, stigma-related barriers and barriers from lack of disability-inclusive training of health care providers. Further studies incorporating disability types in the analysis or focusing on a certain disability type can provide further insights on the type-specific access patterns [7,8,12,39,40]. This will lead to more practical policy implications for tailored healthcare use strategies. Second, the disability subgroup (*n* = 1799) in the study may be underpowered for certain stratified analyses. Non-significant findings in the disability-stratified models, particularly for income, education, and marital status, should be interpreted cautiously as they may have been due to insufficient statistical power. Third, the Korean National Disability Registration System is medically based and mostly captures moderate to severe disabilities. Individuals with mild and unregistered disabilities are not represented in this study, which limits the generalizability of findings to the broader spectrum of disability in Korea. Also, healthcare supply-side factors were not captured in the KNHANES dataset and represent potential sources of residual confounding that could not be controlled in our study. Future studies should incorporate supply-side variables such as availability of specialized providers of people with disabilities to provide a more complete description of UHN determinants in the disability population. Additionally, the study did not include other potential mediators of UHN, such as health literacy, a mediating determinant of health [41,42]. Future studies examining the role of health literacy in UHN among the disability population will be valuable for developing effective and accessible health services and interventions. In addition, as the study is based on a survey, responses are subject to information bias, and expressed need may not strictly align with the clinical definition of healthcare need, which implies the capacity to benefit from healthcare use [43]. Nevertheless, self-reported UHN is a widely used and useful tool for assessing barriers to healthcare access [3,35], and has been reported to be associated with future decline in health outcomes, further supporting its use to assess inequity in healthcare access [2]. To better capture unmet needs for resource allocation and policy design in the disability population, future studies could supplement self-reported UHN data with biomedical measures and health utilization data through linkage of clinical records, enabling a more objective assessment of healthcare needs within the Andersen model framework.

## 5. Conclusions

People with disabilities show significantly higher unmet healthcare needs, and the contribution of predisposing, enabling, and need factors—as conceptualized in Andersen’s Behavioral Model of Health Services Use for vulnerable populations—differs meaningfully by disability status. Interaction term analyses revealed that the effects of sex, a predisposing factor, and household income, an enabling factor, on UHN were significantly moderated by disability status, underscoring that the determinants of healthcare utilization operate differently in the disability population. Furthermore, the differential patterns observed across multiple predisposing, enabling, and need factors suggest that the Andersen model components operate distinctively in the disability population compared to those without disability. These findings carry direct implications for healthcare system planners and policymakers: interventions to reduce UHN in the disability population should move away from uniform strategies and instead adopt a multifactorial, disability-specific approach informed by this framework. Accessible and integrated primary healthcare delivery models can be an effective way of incorporating such approach, which is person-centered and needs-based. Particular attention should be directed toward the most vulnerable subgroups, such as women with disabilities, by implementing female-specific disability healthcare policies such as disability-inclusive services in women’s health services. Also, healthcare delivery systems should prioritize physical accessibility and transport solutions using various approaches from outreach health service programs to incorporating diverse accessible transportation.

## Figures and Tables

**Table 1:** Unmet healthcare need and characteristics of the study population.

Variables	Categories	Total	Without Disability (*n* = 24,634)		With Disability (*n* = 1799)	
			*n*	Percentage	*n*	Percentage
Unmet healthcare need **	No	23,718 (89.7%)	22,157	89.9	1561	86.78
	Yes	2715 (10.3%)	2477	10.1	238	13.2
Predisposing factors **						
Sex **	Male	12,621 (47.7%)	11,515	46.7	1106	61.5
	Female	13,812 (52.3%)	13,119	53.3	693	38.5
Age **	20–34	4847 (18.3%)	4767	19.4	80	4.5
	35–49	7247 (27.4%)	7049	28.6	198	11.0
	50–64	7787 (29.5%)	7206	29.3	581	32.3
	65–79	5735 (21.7%)	4940	20.1	795	44.2
	>80	817 (3.1%)	672	2.7	145	8.1
Marital status **	Married	22,261 (84.2%)	20,616	83.7	1645	91.4
	Other	4172 (15.8%)	4018	16.3	154	8.6
Household size **	1	2908 (11.0%)	2573	10.4	335	18.6
	>1	23,525 (89.0%)	22,061	89.6	1464	81.4
Education **	Primary	5512 (20.9%)	4692	19.1	820	45.6
	Lower-secondary	2729 (10.3%)	2438	9.9	291	16.2
	Higher-secondary	8638 (32.7%)	8178	33.2	460	25.6
	Post-secondary	9554 (36.1%)	9326	37.9	228	12.7
Insurance type **	NHIS	25,616 (96.9%)	24,053	97.6	1563	86.9
	Medicaid	817 (3.1%)	581	2.4	236	13.1
Household Income **	1st quartile	4810 (18.2%)	4061	16.5	754	41.9
	2nd quartile	6464 (24.5%)	5984	24.3	480	26.7
	3rd quartile	7355 (27.8%)	7045	28.6	310	17.2
	4th quartile	7793 (29.5%)	7538	30.6	255	14.2
Paid work **	Yes	16,395 (62.0%)	15,587	63.3	808	44.9
	No	10,038 (38.0%)	9047	36.7	991	55.1
Subjective health **	Very good	1230 (4.7%)	1159	4.7	71	4.0
	Good	6522 (24.7%)	6269	25.5	253	14.1
	Moderate	13,747 (52.0%)	12,963	52.6	784	43.6
	Bad	3951 (14.9%)	3506	14.2	445	24.7
	Very bad	983 (3.7%)	737	3.0	246	13.7
Obesity **	No	17,196 (65.1%)	16,149	65.6	1047	58.2
	Yes	9237 (34.9%)	8485	34.4	752	41.8
Hypertension **	No	11,434 (43.3%)	10,959	44.5	475	26.4
	Prehypertension	6442 (24.4%)	6014	24.4	428	23.8
	Hypertension	8557 (32.4%)	7661	31.1	896	49.8
Diabetes **	No	16,369 (61.9%)	15,533	63.1	836	46.5
	Prediabetes	6696 (25.3%)	6192	25.1	504	28.0
	Diabetes	3368 (12.7%)	2909	11.8	459	25.5
Hypercholesterolemia **	No	20,945 (79.2%)	19,637	79.7	1308	72.7
	Yes	5488 (20.8%)	4997	20.3	491	27.3
Functional Limitations **	No	24,330 (92.0%)	23,035	93.5	1295	72.0
	Yes	2103 (8.0%)	1599	6.5	504	28.0
Mobility **	No problem	22,764 (86.1%)	21,656	87.9	1108	61.6
	Some problem	3501 (13.2%)	2862	11.6	639	35.5
	Extreme problem	168 (0.6%)	116	0.5	52	2.9
Usual activity **	No problem	24,323 (92.0%)	23,020	93.5	1303	72.4
	Some problem	1995 (7.5%)	1544	6.3	451	25.1
	Extreme problem	115 (0.4%)	70	0.3	45	2.5

** *p* < 0.01, *p*-values derived by chi-square analysis between disability status groups.

**Table 2:** Differences of unmet healthcare needs of people without and with disability depending on their characteristics (chi-square analysis).

		Unmet Healthcare Need (*n*, %)			
Variables	Categories	Without Disability (*n* = 24,634)	*p*-Value	With Disability (*n* = 1799)	*p*-Value
Predisposing Factors					
Sex	M	872 (7.6%)	<0.001 **	98 (8.9%)	<0.001 **
	F	1605 (12.2%)		140 (20.2%)	
Age	20–34	527 (11.1%)	<0.001 **	11 (13.7%)	0.201
	35–49	641 (9.1%)		19 (9.6%)	
	50–64	690 (9.6%)		73 (12.6%)	
	65–79	531 (10.8%)		120(15.1%)	
	>80	88 (13.1%)		15 (10.3%)	
Marital status	Married	2066 (10.0%)	0.689	220 (13.4%)	0.555
	other	411 (10.2%)		18 (11.7%)	
Household size	1	391 (15.2%)	<0.001 **	55 (16.4%)	0.056
	>1	2086 (9.5%)		183 (12.5%)	
Education	Primary	696 (14.8%)	<0.001 **	143 (17.4%)	<0.001 **
	Lower-secondary	249 (10.2%)		30 (10.3%)	
	Higher-secondary	744 (10.1%)		44 (9.6%)	
	Post-secondary	788 (8.4%)		21 (9.2%)	
Enabling Factors					
Insurance type	NHIS	2358 (9.8%)	<0.001 **	193 (12.4%)	0.005 **
	Medicaid	119 (20.5%)		45 (19.1%)	
Household income	1st quartile	627 (15.4%)	<0.001 **	118 (15.7%)	0.060
	2nd quartile	642 (10.7%)		52 (10.8%)	
	3rd quartile	638 (9.1%)		40 (12.9%)	
	4th quartile	570 (7.6%)		28 (11.0%)	
Paid work	Yes	1567 (10.1%)	0.989	95 (11.8%)	0.096
	No	910 (10.1%)		143 (14.4%)	
Need Factors					
Subjective health	Very good	49 (4.2%)	<0.001 **	1 (1.4%)	<0.001 **
	Good	337 (5.4%)		16 (6.3%)	
	Moderate	1236 (9.5%)		80 (10.2%)	
	Bad	655 (18.7%)		75 (16.9%)	
	Very bad	200 (27.1%)		66 (26.8%)	
Obesity	No	1627 (10.1%)	0.887	123 (11.8%)	0.029 *
	Yes	850 (10.0%)		115 (15.3%)	
Hypertension	No	1191 (10.9%)	<0.001 **	64 (13.5%)	0.839
	Prehypertension	548 (9.1%)		53 (12.4%)	
	Hypertension	738 (9.6%)		121 (13.5%)	
Diabetes	No	1599 (10.3%)	0.033 *	124 (14.8%)	0.142
	Prediabetes	570 (9.2%)		63 (12.5%)	
	Diabetes	308 (10.6%)		51 (11.1%)	
Hypercholesterolemia	No	1955 (10.0%)	0.303	175(13.4%)	0.760
	Yes	522 (10.5%)		63(12.8%)	
Functional limitations	Yes	361 (22.6%)	<0.001 **	106 (21.0%)	<0.001 **
	No	2116 (9.2%)		132 (10.2%)	
Mobility	No problem	1851 (8.6%)	<0.001 **	97 (8.7%)	<0.001 **
	Some problem	580 (20.3%)		120 (18.8%)	
	Extreme problem	46 (39.7%)		21 (40.4%)	
Usual activity	No problem	2057 (8.9%)	<0.001 **	134 (10.3%)	<0.001 **
	Some problem	396 (25.7%)		91 (20.2%)	
	Extreme problem	24 (34.3%)		13 (28.9%)	

* *p* < 0.05, ** *p* < 0.01.

**Table 3:** Multiple logistic regression model on the self-reported unmet healthcare needs with interaction terms.

		Model 1		Model 2	
Variables		aOR	95% CI	aOR	95% CI
Predisposing Factors					
Vulnerability domain					
Disability (ref: No)	Yes	1.21 *	(1.04–1.41)	0.90	(0.77–1.06)
Traditional domain					
Sex (ref: Male)	Female	1.67 **	(1.53–1.82)	1.50 **	(1.37–1.64)
Age (ref: >80)	20–34	2.15 **	(1.62–2.84)	2.48 **	(1.83–3.34)
	35–49	1.51 *	(1.17–1.95)	1.76 **	(1.34–2.32)
	50–64	1.26	(0.99–1.60)	1.49 *	(1.16–1.92)
	65–79	1.05	(0.84–1.32)	1.18	(0.93–1.49)
Marital status (ref: other)	Married	1.17 *	(1.00–1.36)	1.17 *	(1.01–1.37)
Household size (ref: >1)	1	1.29 **	(1.14–1.46)	1.23 **	(1.09–1.40)
Education (ref: Post-secondary)	Primary	1.85 **	(1.59–2.15)	1.42 **	(1.21–1.66)
	Lower-secondary	1.31 *	(1.11–1.54)	1.13	(0.96–1.34)
	Higher-secondary	1.08	(0.97–1.20)	1.04	(0.94–1.16)
Enabling Factors					
Traditional/Vulnerability domain					
Insurance type (ref: NHIS)	Medicaid	1.56 **	(1.29–1.90)	1.16	(0.95–1.42)
Household income (ref: 4th quartile)	1st quartile	1.78 **	(1.54–2.05)	1.51 **	(1.31–1.75)
	2nd quartile	1.33 **	(1.18–1.50)	1.26 **	(1.12–1.43)
	3rd quartile	1.17 *	(1.04–1.32)	1.13 *	(1.01–1.28)
Paid work (ref: Yes)	No	1.30 **	(1.18–1.42)	1.49 **	(1.35–1.64)
Need Factors					
Traditional domain					
Subjective health (ref: Very good)	Good			1.31	(0.96–1.77)
	Moderate			2.29 **	(1.71–3.06)
	Bad			4.04 **	(2.99–5.45)
	Very bad			4.56 **	(3.26–6.39)
Obesity (ref: No)	Yes			1.03	(0.94–1.13)
Hypertension (ref: No)	Prehypertension			0.84 *	(0.75–0.94)
	Hypertension			0.73 **	(0.65–0.82)
Diabetes (ref: No)	Prediabetes			0.95	(0.86–1.06)
	Diabetes			0.85 *	(0.74–0.98)
Hypercholesterolemia (ref: No)	Yes			0.95	(0.85–1.06)
Vulnerability domain					
Functional limitations (ref: No)	Yes			1.41 **	(1.23–1.62)
Mobility (ref: No problem)	Some problem			1.61 **	(1.41–1.84)
	Extreme problem			2.72 **	(1.89–3.93)
Usual activity (ref: No problem)	Some problem			1.45 **	(1.25–1.69)
	Extreme problem			1.57 **	(1.00–2.45)
Interaction terms (Model 2 only)					
Interaction term: Disability × sex				1.61 *	(1.20–2.16)
Interaction term: Disability × household income				0.58 **	(0.43–0.78)

* *p* < 0.05, ** *p* < 0.01, aOR = adjusted odds ratio; CI = confidence interval; ref = reference category. Model 1 runs on the predisposing and enabling variables; Model 2 includes predisposing, enabling, and need variables. Interaction terms tested using the likelihood ratio test.

**Table 4:** Multiple logistic regression model on the self-reported unmet health care needs stratified by disability status.

		Without Disability		With Disability	
Variables		aOR	(95% CI)	aOR	(95% CI)
Predisposing factors					
Sex (ref: Male)	Female	1.44 **	(1.31–1.59)	2.21 **	(1.61–3.03)
Age (ref: >80)	20–34	2.42 **	(1.76–3.34)	2.83	(0.94–8.54)
	35–49	1.74 **	(1.30–2.34)	1.68	(0.71–3.99)
	50–64	1.44 *	(1.09–1.90)	2.02 *	(1.03–3.94)
	65–79	1.09	(0.84–1.41)	1.98 *	(1.07–3.66)
Marital status (ref: Other)	Married	1.19 *	(1.02–1.40)	1.01	(0.51–2.00)
Household size (ref: >1)	1	1.26 **	(1.10–1.44)	1.02	(0.69–1.50)
Education (ref: Post-secondary)	Primary	1.38 *	(1.17–1.63)	1.49	(0.82–2.72)
	Lower-secondary	1.15	(0.96–1.37)	0.96	(0.50–1.84)
	Higher-secondary	1.04	(0.93–1.12)	0.98	(0.55–1.75)
Enabling factors					
Insurance type (ref: NHIS)	Medicaid	1.14	(0.9–1.44)	1.57	(0.99–2.48)
Household income (ref: 4th quartile)	1st quartile	1.62 **	(1.39–1.88)	0.76	(0.45–1.29)
	2nd quartile	1.31 **	(1.15–1.48)	0.72	(0.42–1.22)
	3rd quartile	1.14 *	(1.01–1.28)	1.04	(0.60–1.79)
Paid work (ref: Yes)	No	1.48 **	(1.34–1.64)	1.53 *	(1.09–2.16)
Need factors					
Subjective health (ref: Very good)	Good	1.24	(0.91–1.69)	5.10	(0.65–40.02)
	Moderate	2.19 **	(1.63–2.94)	8.23 *	(1.10–61.48)
	Bad	3.94 **	(2.91–5.34)	10.88 *	(1.45–81.95)
	Very bad	4.22 **	(2.97–6.01)	16.22 *	(2.13–123.54)
Obesity (ref: No)	Yes	0.98	(0.90–1.07)	1.16	(0.88–1.53)
Hypertension (ref: No)	Prehypertension	0.83 *	(0.74–0.93)	0.94	(0.62–1.43)
	Hypertension	0.71 **	(0.63–0.81)	0.88	(0.60–1.30)
Diabetes (ref: No)	Prediabetes	0.96	(0.86–1.07)	0.88	(0.62–1.26)
	Diabetes	0.91	(0.78–1.05)	0.58 *	(0.39–0.85)
Hypercholesterolemia (ref: No)	Yes	0.98	(0.87–1.10)	0.76	(0.54–1.06)
Functional limitations (ref: No)	Yes	1.39 **	(1.19–1.62)	1.53 *	(1.10–2.15)
Mobility (ref: No problem)	Some problem	1.61 **	(1.39–1.86)	1.60 *	(1.01–2.34)
	Extreme problem	2.54 **	(1.65–3.90)	3.65 **	(1.73–7.69)
Usual activity (ref: No problem)	Some problem	1.55 **	(1.32–1.83)	1.12	(0.76–1.65)
	Extreme problem	1.74	(1.00–3.02)	1.26	(0.56–2.84)

* *p* < 0.05, ** *p* < 0.01, aOR = adjusted odds ratio; CI = confidence interval; ref = reference category.

## Data Availability

Although the original KNHANES data are publicly available in KNHANES website at “https://knhanes.kdca.go.kr (accessed on 18 April 2026)”, the data for this study have been exclusively prepared by linking the original data with NHIS patient status data through the Korea Healthcare Bigdata platform. Due to the data security policy of the platform, the datasets generated are not readily available to the public.

## Abbreviations

The following abbreviations are used in this manuscript:

UHC	Universal Health Coverage
UHN	Unmet healthcare needs
KNHANES	Korea National Health and Nutritional Examination Survey
NHIS	National Health Insurance Service
